# PHF20 positively regulates osteoblast differentiation via increasing the expression and activation of Runx2 with enrichment of H3K4me3

**DOI:** 10.1038/s41598-017-08868-0

**Published:** 2017-08-14

**Authors:** Jin-Woo Yang, Byung-Chul Jeong, Jongsun Park, Jeong-Tae Koh

**Affiliations:** 1 Department of Pharmacology and Dental Therapeutics, School of Dentistry, Chonnam National University, Gwangju, 61186 South Korea; 2 Research Center for Biomineralization Disorders, School of Dentistry, Chonnam National University, Gwangju, 61186 South Korea; 3 Department of Pharmacology and Medical Science, College of Medicine, Chungnam National University, Daejeon, 35015 South Korea

## Abstract

Plant homeodomain finger protein 20 (PHF20), a methyl lysine effector protein, is a component MOF-NSL lysine acetyltranferase complex. Global deletion of PHF20 has shown spinal bone defects and reduced skeletal formation. However, the molecular basis of PHF20 involved in skeletal development has not been elucidated yet. The objective of this study was to determine the role of PHF20 in osteoblast differentiation and mineralization. Expression of PHF20 was gradually increased during osteoblast differentiation. Overexpression of PHF20 enhanced ALP activity and mineralized nodule formation as well as the expression of osteogenic markers including Runx2. In contrast, inhibition of PHF20 expression reduced osteoblast differentiation and mineralization. Mechanistically, PHF20 increased the promoter activity of osteogenic genes including Og2, Alp, and Bsp through direct association with Runx2. Moreover, PHF20 increased the enrichment of H3K4me3 on the promoter of Runx2 followed by increased Runx2 promoter activity. Interestingly, Bix-01294, a histone methylation inhibitor, decreased mineralized nodule formation through decreasing the levels of H3K4me3 and Runx2. Overexpression of PHF20 restored the Bix-01294 effects. Taken together, these results indicate that methyl lysine-binding protein PHF20 might be a novel regulator of osteoblast differentiation.

## Introduction

Osteoblast differentiation and bone formation are regulated by a number of extracellular molecules such as bone morphogenetic protein 2 (BMP2), transcriptional factors, and posttranslational modifiers. Transcriptional factor runt-related transcription factor 2 (Runx2) is a key regulator of osteoblast differentiation^1–4^. Mutation of Runx2 can result in the formation of skeletal parts such as cleidocranial dysplasia^5, 6^. Runx2 null mice are found to have defects in skeletal formation due to maturational arrest of osteoblast differentiation^5, 7^.

Runx2 has two isoforms: Runx2-II and Runx2-I. They have different amino terminal sequences. The production of Runx2-II and Runx2-I is controlled by a distal promoter (P1) and a proximal promoter (P2), respectively^8^. Runx2 P1 promoter transcript is more relevant to bone than the P2 promoter. It is active in mature osteoblasts and hypertrophic chondrocytes^9^. The production of Runx2 transcript in osteoblast differentiation is affected by various post-translational modifications (PTMs) of histone, including methylation, acetylation, and phosphorylation^10, 11^. In general, histone modification is catalyzed by several PTM enzymes such as histone methyltransferases (HMTases) and histone acetyltransferases (HATases) with completely different transcriptional outputs and biological functions depending on the specific genomic loci or chromosomal domains beyond protein expression according to nucleotide sequence^12, 13^. Osteoblast specific gene transcription and differentiation are activated by methylation or acetylation of histone 3 lysine 4 (H3K4). While, methylation of histone 3 lysine 9 (H3K9) and histone 3 lysine 27 (H3K27) inhibited the gene activity^14^.

Plant homeodomain finger protein 20 (PHF20) has multiple domains. It is a methyl lysine effector. It is also a component of males absent on the first (MOF)-nonspecific lethal (NSL) lysine acetyltransferase complex involved in the acetylation of histone and non-histone proteins^15–17^. PHF20 has two types of domains (two N-terminal Tudor domains and one C-terminal PHD finger domain) that can bind methylated residues^18^. Recent studies have shown that the second Tudor domain of PHF20 is dimerized and binds directly to p53K370me2 and p53K382me2, which enhances the binding of PHF20 to p53. In addition, PHF20 acts as a methylation effector that contributes to the up-regulation of p53 in response to DNA damage, and thereby plays a role in the activation and stabilization of p53 protein^16, 19^. PHF20 is involved in histone 4 lysine 16 (H4K16) acetylation as a member of the MOF-NSL complex. PHD finger domain can recognize H3K4me2 residues and affect its methylation along with mixed lineage leukemia 1 (MLL1)-lysine methyltransferase (KMT) complex^16, 20^.

PHF20 affects cell growth, differentiation, and survival through epigenetic modification of the target gene^16^. In addition, it has been reported that PHF20 knock-out (KO) mouse has a weak skeleton with lumbar vertebrae missing^15^. Bone density analysis through microCT has shown that PHF20 KO mice have lower skeleton level compared to WT mice^15^. Overall, the lack of PHF20 had led to abnormal development of skeletal formation in mice. However, the role of PHF20 in osteoblast differentiation has not been reported yet.

Thus, the objective of this study was to determine the expression profile of PHF20 during osteoblast differentiation and alteration of the PHF20 effects by overexpressing or inhibiting PHF20. Our results revealed that PHF20 could regulate osteoblast differentiation by altering the level of histone methylation at the promoter of Runx2 and the level of Runx2 transcript. In addition, PHF20 interacted with Runx2 to stimulate Runx2 activity. Our data provided a clue that PHF20 might be a novel regulator of osteoblast differentiation.

## Results

### PHF20 expression increased during osteoblast differentiation

**Figure 1:**
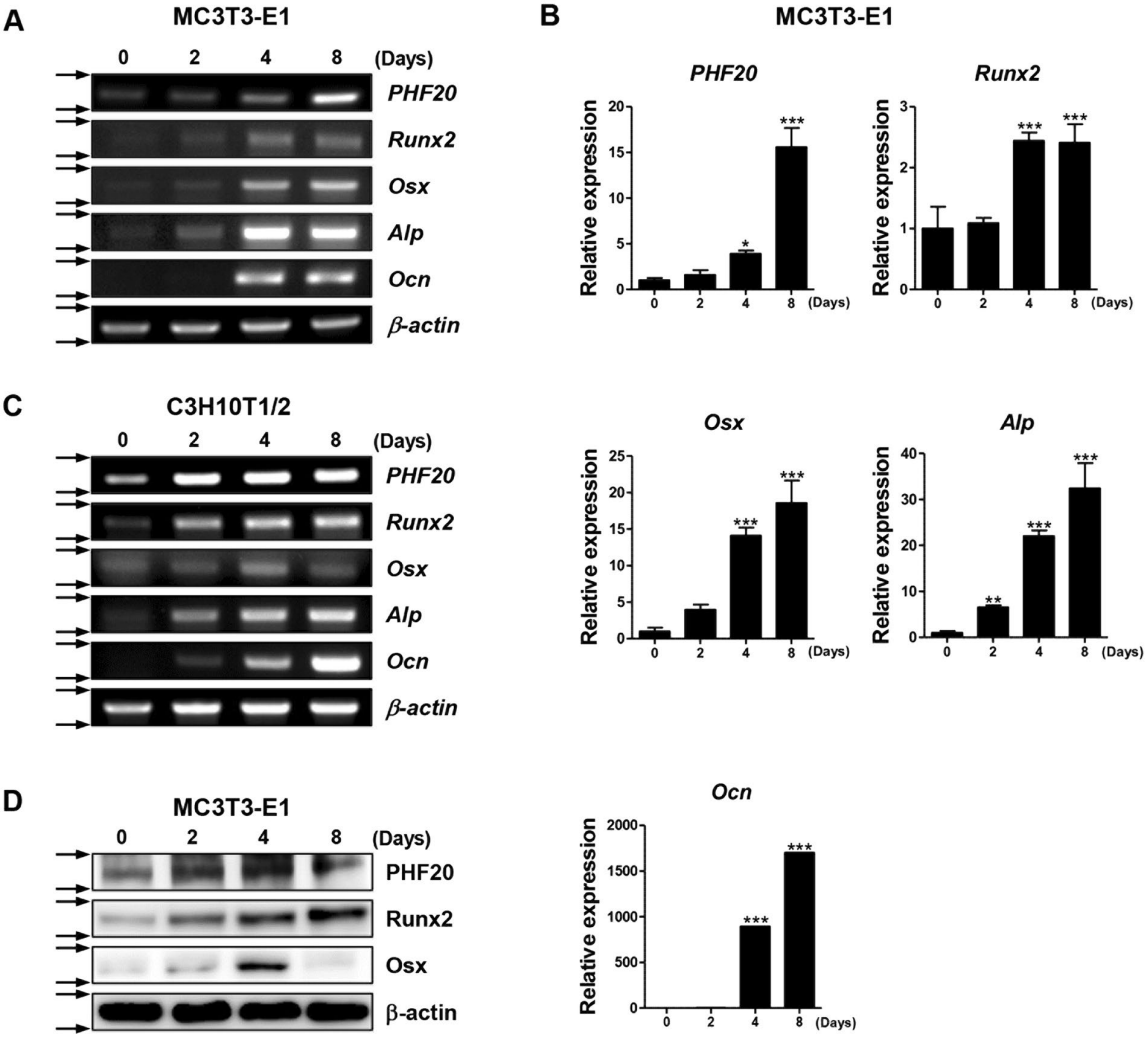
Expression profiles of PHF20 during osteoblast differentiation. Osteoblast lineage MC3T3-E1 and C3H10T1/2 cells were differentiated in osteogenic medium (OM) for 0, 2, 4, and 8 days. Expression levels of PHF20 and osteoblast differentiation markers were determined by RT-PCR (**A**,**C**), qRT-PCR (**B**), and Western blot (**D**). β-actin was used as internal control. Arrows (→) (**A**,**C**,**D**) indicate cropping lines and gels/blots were obtained under the same experimental conditions. Values are expressed as means ± SEM (n = 3). *p < 0.05, **p < 0.01 and ***p < 0.001 compared to Day 0.

To examine whether PHF20 has a certain role in osteoblast differentiation, levels of PHF20 mRNA and protein were examined during osteoblastic differentiation of pre-osteoblast lineage MC3T3-E1 cells. The cells were cultured in osteogenic medium (OM) for indicated time, and then RT-PCR and quantitative real-time PCR (qRT-PCR) analyses were performed. Results showed that mRNA expressions of osteoblast-specific genes, including osterix (Osx), alkaline phosphatase (Alp), osteocalcin (Ocn), and PHF20, gradually increased up to 8 days after induction. Runt-related transcription factor 2 (Runx2) mRNA expression was peaked at 4 days without more increasing at 8 days (Fig. 1A,B). In the osteoblastic differentiation of pluripotent mesenchymal lineage C3H10T1/2 cells, level of PHF20 mRNA also increased with the increases in Alp, Ocn, and Runx2 mRNA expressions (Fig. 1C). Western blot analysis confirmed the increases of expression of PHF20 and Runx2 at protein level. The expression level of Osx protein was also increased (Fig. 1D). These results show an association of PHF20 in osteoblast differentiation.

### Overexpression of PHF20 accelerates osteoblast differentiation

**Figure 2:**
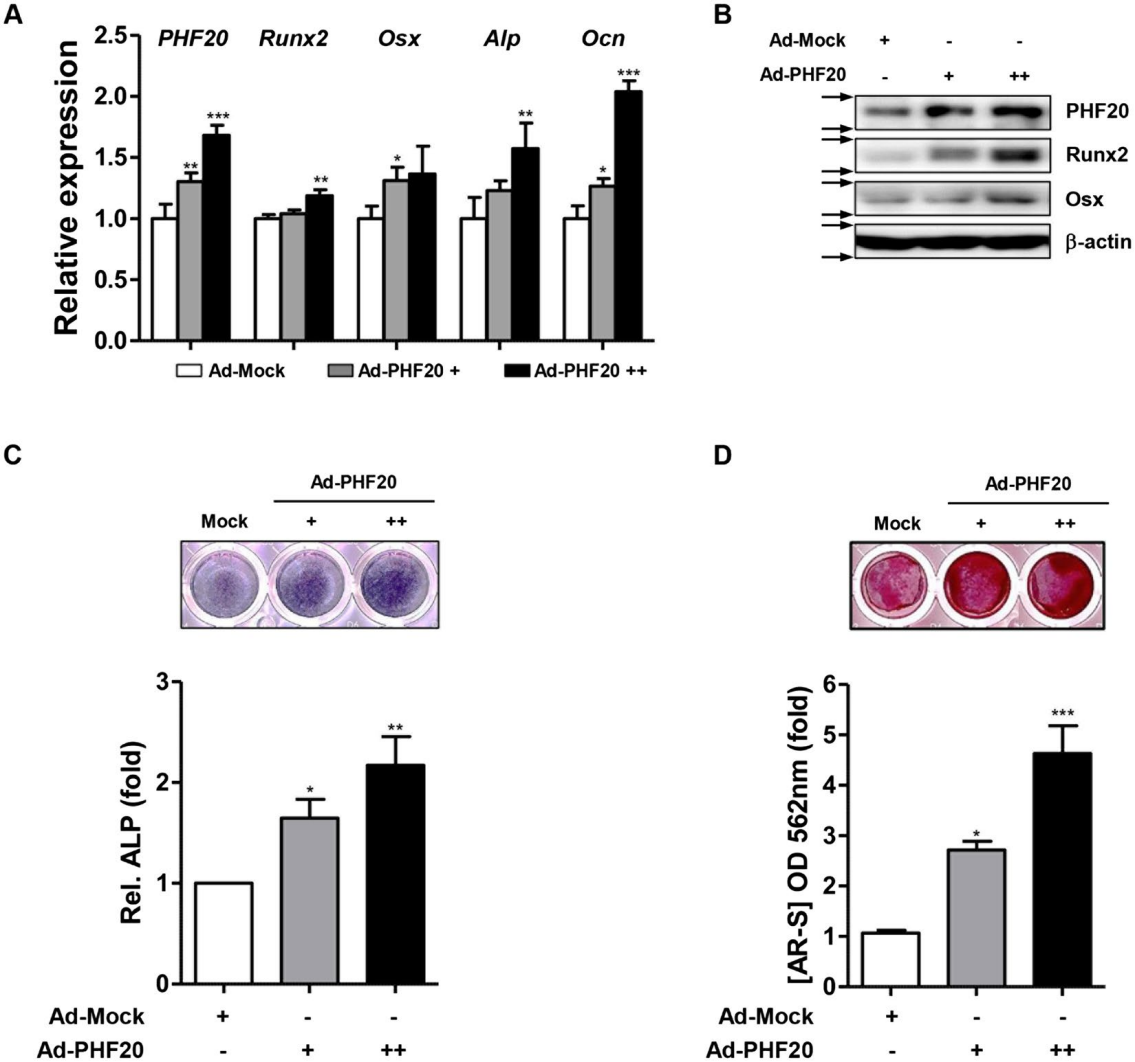
Overexpression of PHF20 promotes osteoblast differentiation. MC3T3-E1 cell were infected with Ad-PHF20 (+, 50 MOI; ++, 100 MOI) or Ad-GFP (+, 50 MOI) as Mock and cultured for 24 hours with OM for qRT-PCR (**A**). Western blot analysis (**B**). At 24 hours after adenovirus infection, cells were cultured with OM for ALP staining (**C**) and alizarin red staining (AR-S) (**D**). Arrows (→) indicate cropping lines and blots were obtained under the same experimental conditions (**B**). ALP staining was performed with BCIP/NBT solution at 3 days. AR-S was performed at 8 days. For quantitation, ALP stained cells were quantified using ImageJ software (**C**, lower). Alizarin red stained cells were extracted with 10% (w/v) cetylpyridinium chloride and the absorbance value at 562 nm was then measured by spectrophotometry (**D**, lower). Values are expressed as means ± SEM (n = 3). *p < 0.05, **p < 0.01 and ***p < 0.001 compared to Ad-Mock.

In order to investigate the role of PHF20 in osteoblast differentiation, effects of PHF20 overexpression on the expression of osteoblast-specific genes, ALP enzyme activity, and matrix mineralization in MC3T3-E1 cells were examined using an adenovirus encoding for PHF20 (Ad-PHF20). Overexpression of PHF20 increased the mRNA levels of Runx2, Osx, Alp, and Ocn based on qRT-PCR (Fig. 2A, Supplementary Fig. 1). Overexpression of PHF20 also increased the protein levels of Runx2 and Osx based on Western blot analysis (Fig. 2B). In addition, when MC3T3-E1 cells were cultured with osteogenic induction media for 3 days and 8 days, overexpression PHF20 increased ALP activity (Fig. 2C) and calcium deposition (Fig. 2D) in a dose-dependent manner using an ALP staining kit and alizarin red stain (AR-S). These results suggest that PHF20 can stimulate osteoblast differentiation.

### Knockdown of PHF20 reduces osteoblast differentiation

**Figure 3:**
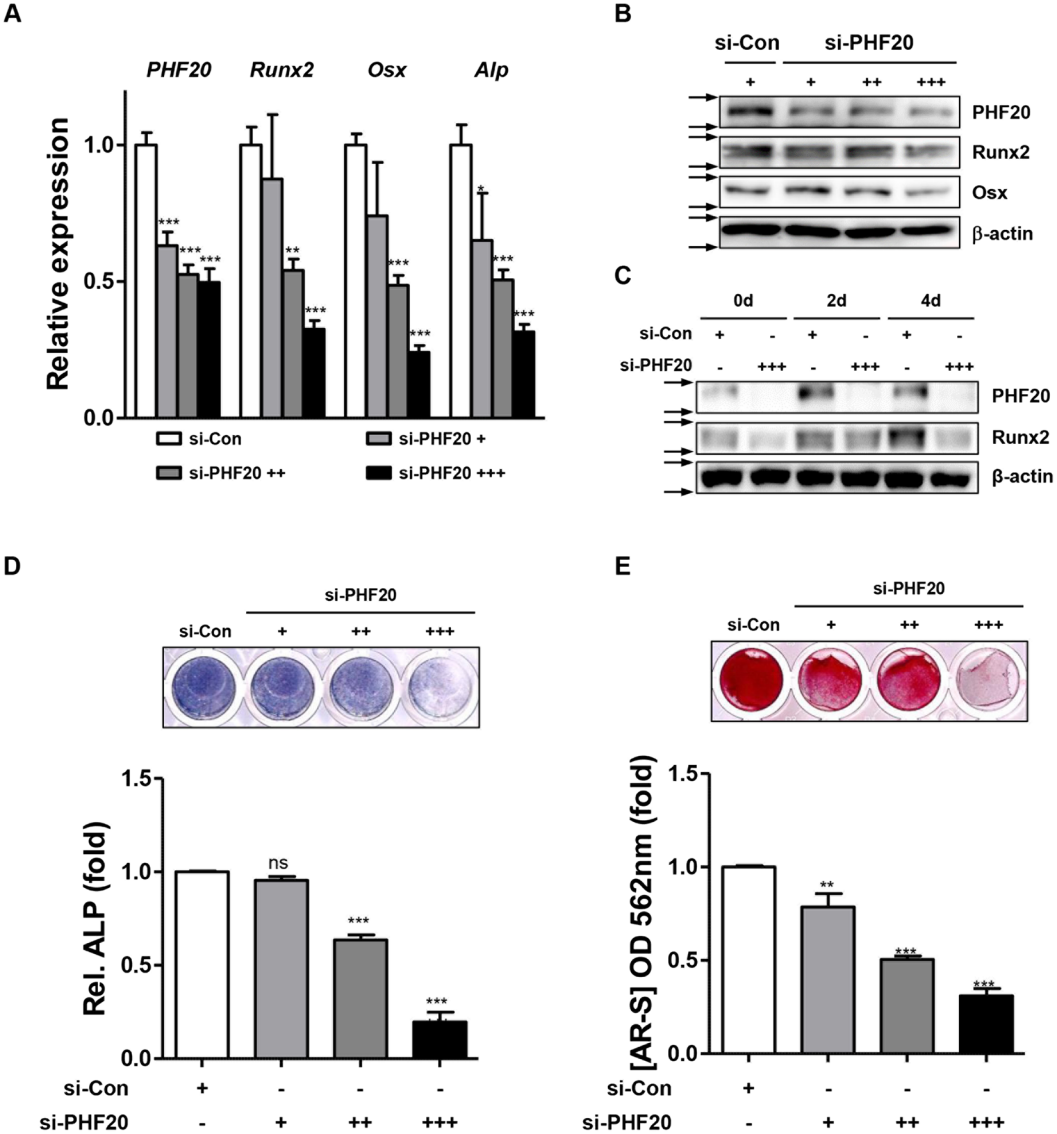
Knockdown of PHF20 reduces osteoblast differentiation. Cells were transfected with si-Control and si-PHF20 (+, 30 nM; ++, 60 nM; +++, 90 nM) for 2 days. mRNA and proteins were extracted for qRT-PCR (**A**) and Western blot analyses (**B**). For time course effects, cells were treated with si-PHF20 (90 nM) for 4 days, and Western blot analysis was performed (**C**). Arrows (→) indicate cropping lines and blots were obtained under the same experimental conditions (**B**,**C**). Cells were stained for ALP at day 3 (**D**), AR-S was performed at day 8 of culture (**E**). For quantitation, ALP stained cells were quantified using ImageJ software (**D**, lower). Alizarin red stained cells were extracted with 10% cetylpyridinium chloride and the absorbance value was measured at 562 nm by spectrophotometry (**E**, lower). Values are expressed as means ± SEM (n = 3). **p < 0.01 and ***p < 0.001 compared to si-Con.

To ascertain the stimulatory effect of PHF20 on osteoblast differentiation, we performed loss-of-PHF20 function experiment using si-RNA specific for PHF20 (si-PHF20) in MC3T3-E1 cells. Treatment with si-PHF20 dose-dependently decreased PHF20 expression. It also decreased Runx2 and Osx expression at mRNA (Fig. 3A) and protein level (Fig. 3B). Treatment of si-PHF20 (90 nM) suppressed osteogenic medium induction of PHF20 and Runx2 protein expression up to 4 days (Fig. 3C). ALP staining and alizarin red stain results showed that inhibition of PHF20 expression consistently and dose-dependently decreased ALP enzyme activity and calcium deposition (Fig. 3D,E). These results consistently support the notion that PHF20 plays a stimulatory role in osteoblast differentiation.

### PHF20 stimulates osteoblast differentiation via physical interaction with Runx2

**Figure 4:**
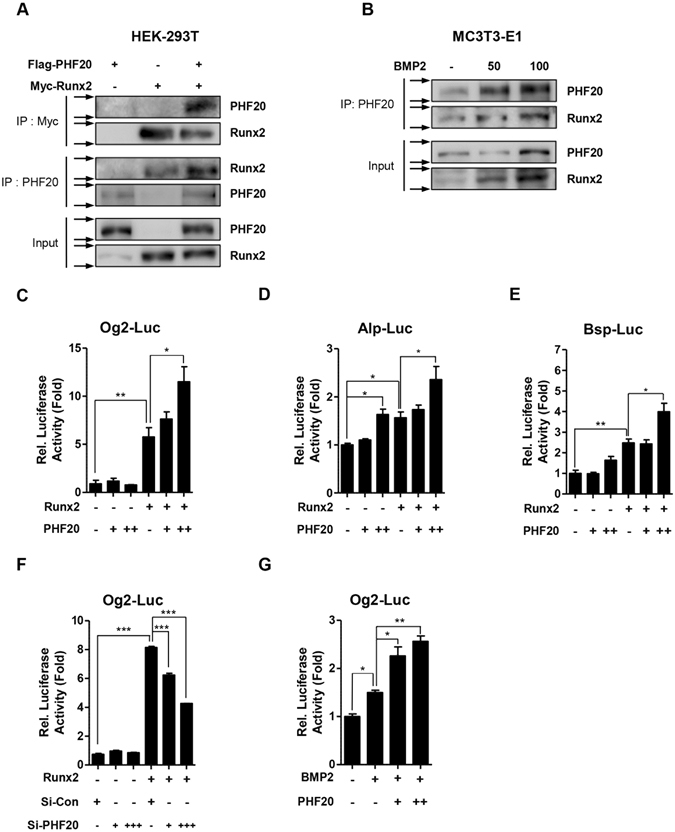
PHF20 regulates Runx2 transactivity through physical interaction with Runx2. HEK-293T cells were transiently transfected with Flag-PHF20 and Myc-Runx2 for 48 hours. Immunoprecipitation was performed with Myc or PHF20 antibody followed by immunoblotting with PHF20 and Runx2 antibody (**A**). MC3T3-E1 cells were treated with BMP2 for 3 days and immunoprecipitated to confirm endogenous protein binding (**B**). Arrows (→) indicate cropping lines and blots were obtained under the same experimental conditions (**A**,**B**). PHF20 regulates luciferase activities of osteoblast specific marker genes (**C**–**G**). MC3T3-E1 cells were transiently transfected with Og2-Luc (200 ng) (**C**), Alp-Luc (200 ng) (**D**), Bsp-Luc reporter (200 ng) (**E**), plasmid with Runx2 (100 ng) construct and/or PHF20 (+, 200 ng; ++, 600 ng) construct. Luciferase activities of Og2-Luc by si-Control and si-PHF20 (+, 30 nM; +++, 90 nM) treatment were measured (**F**). Luciferase activities of Og2-Luc were increased by BMP2 (100 ng/ml) and PHF20 (+, 200 ng; ++, 600 ng) (**G**). At 12 hours after transfection, cells were treated with or without BMP2 for 48 hours. After transfection, luciferase assay was performed. Results are expressed as fold activity relative to the control (**C**–**G**). Values are means ± SEM (n = 3). *p < 0.05, **p < 0.01 and ***p < 0.001 compared to the indicated group.

Runx2 is the master transcriptional factor for controlling osteoblast differentiation, and the factor complicatedly regulates osteoblast differentiation via cooperating with other regulatory proteins^4, 21^. To determine whether PHF20 could interact with Runx2 to stimulate osteoblast differentiation, immunoprecipitation assay was performed. HEK-293T cells were transfected with Flag-tagged PHF20 or Myc-tagged Runx2 constructs. Cell lysates were then immunoprecipitated with Myc or PHF20 antibodies followed by Western blot analyses were using PHF20 and Runx2 antibodies. As shown in Fig. 4A, exogenously overexpressed PHF20 and Runx2 could physically interact with each other in cells. When pre-osteoblast MC3T3-E1 cells were treated with osteogenic BMP2, PHF20 and Runx2 expression were increased by BMP2 in a dose-dependent manner (Fig. 4B, lower panel). Immunoprecipitation assay revealed that the interaction between PHF20 and Runx2 was also increased (Fig. 4B, upper panel), suggesting that endogenous PHF20 could interact with endogenous Runx2 in osteoblast lineage cells.

This study further examined the effects of PHF20 on Runx2 activity in MC3T3-E1 cells using Runx2 binding osteoblast-specific genes with luciferase reporter. Overexpression of PHF20 dose-dependently enhanced Runx2-induced luciferase activity in MC3T3-E1 cells transfected with Osteocalcin gene 2 promoters-Luciferase (Og2-Luc), Alkaline phosphatase promoters-Luciferase (Alp-Luc) or Bone sialoprotein promoters-Luciferase (Bsp-Luc) construct (Fig. 4C–E). Overexpression of PHF20 alone did not significantly increase the luciferase activity of Og2-Luc plasmid containing cells (Fig. 4C). However, in cells containing the artificial six-copy osteoblast-specific cis-acting element- Luciferase (6xOSE-Luc), the luciferase activity was increased (Supplementary Fig. 2). On the contrary, inhibition of PHF20 expression using si-RNA dose-dependently inhibited Runx2-induced luciferase activity in Og2-Luc treated cells (Fig. 4F). After treatment with BMP2 to stimulate Runx2 expression^3, 22, 23^, PHF20 overexpression also dose-dependently increased the activity of Og2-Luc (Fig. 4G). These results suggest that PHF20 could regulate osteoblast-specific gene expression via interacting with Runx2.

### PHF20 mediates enrichment of H3K4me3 at the Runx2 P1 promoter accompanied by Runx2 expression in osteoblasts

**Figure 5:**
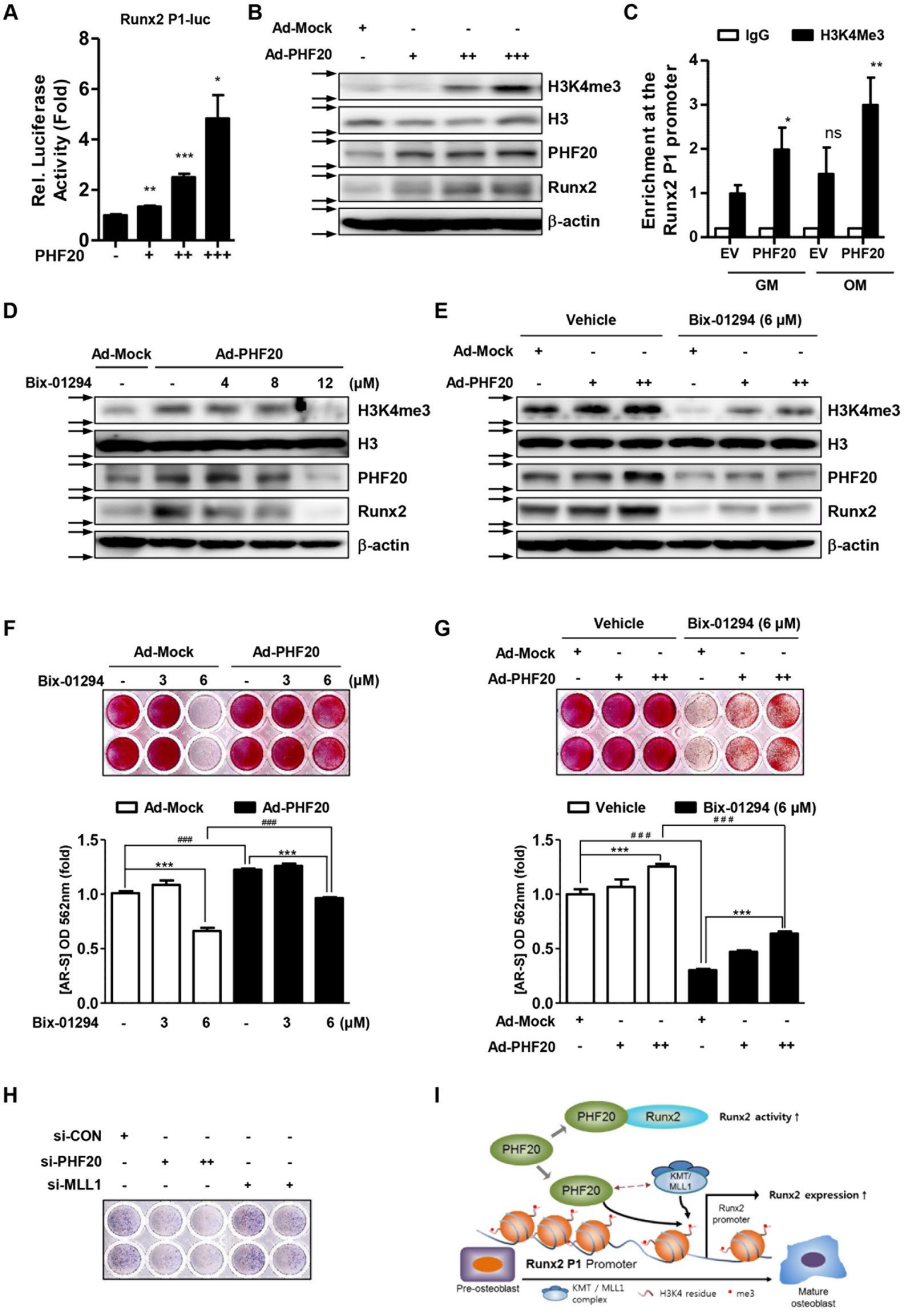
PHF20 increases enrichment of H3K4me3 at the P1 promoter of Runx2 accompanied by Runx2 expression. (**A**) Effects of PHF20 on luciferase activity of P1 Promoter of Runx2. MC3T3-E1 cells were co-transfected with Runx2-756-P1-Luc (200 ng) and PHF20 expression vectors (+, 100 ng; ++, 200 ng; +++, 300 ng). Values are means ± SEM (n = 3). *p < 0.05, **p < 0.01 and ***p < 0.001 compared to the control. (**B**) Effects of PHF20 on H3K4me3 levels. MC3T3-E1 cells were infected by Ad-GFP (+, 50 MOI) as a mock, Ad-PHF20 (+, 20 MOI; ++, 50 MOI; +++, 100 MOI), and then Western blot analysis was performed. (**C**) Chromatin immunoprecipitation (ChIP) analysis of the recruitment of H3K4me3 to Runx2-756-P1 promoter region. Transfected whole cell lysates were precipitated using antibodies against H3K4me3 or IgG. PCR was performed to quantify binding of the 500 bp of the P1 promoter of Runx2 to H3K4me3. IgG was used as a specificity control. Values are means ± SEM (n = 3). *p < 0.05 and **p < 0.01 compared to cells transfected with empty vector (E.V). (**D**,**E**) Combinatory effects of Bix-01294 and PHF20 overexpression on H3K4me3 and Runx2 protein levels. Cells were treated with treatment of Ad-Mock (50 MOI) and Ad-PHF20 (100 MOI) for 24 hours followed by the indicated amount of Bix-01294 treatment for 72 hours. Arrows (→) indicate cropping lines and blots were obtained under the same experimental conditions. (**F**,**G**) Combinatory effects of Bix-01294 and PHF20 overexpression on mineralization nodule formation. Cells were treated with Bix-01294 and PHF20 for 8 days, and AR-S was performed. Alizarin red stained cells were extracted with 10% (w/v) cetylpyridinium chloride and the absorbance value at 562 nm was then measured by spectrophotometry. Values are means ± SEM (n = 3). ***p < 0.001 and ^###^P < 0.001 compared to the indicated group. (**H**) Effects of si-MLL1 on ALP activity. Cells were cultured with osteogenic medium including si-control, si-PHF20 (+, 30 nM; ++, 90 nM) or si-MLL1 (+, 50 nM; ++, 200 nM) for 3 days. (**I**) Proposed model describing the regulation of Runx2 by PHF20 in osteoblast differentiation.

The methyl lysine effector protein PHF20 can regulate the expression levels of several genes with the increases in protein methylation such as p53^16, 19^. Methylation of histone H3K4 on Runx2 promoter region can alter Runx2 expression and osteoblastogenesis^11, 24^. In this study, we examined the effect of PHF20 on Runx2 promoter activity and methylation of H3K4. PHF20 overexpression dose-dependently increased the P1 promoter activity of Runx2 (Fig. 5A). It also dose-dependently increased the protein expression level of Runx2 and tri-methylated form of H3K4 in MC3T3-E1 cells (Fig. 5B). In addition, chromatin immunoprecipitation assay showed that PHF20 overexpression increased the enrichment of H3K4me3 on the P1 promoter region of Runx2 (Fig. 5C). On the contrary, the histone lysine methyltransferase inhibitor Bix-01294 attenuated the effects (increased H3K4 methylation, increased Runx2 expression, and increased calcium deposition) induced by PHF20 (Fig. 5D,F). Overexpression of PHF20 following treatment with 6 μM Bix-01294 partially recovered the decreased H3K4me3 and Runx2 levels (Fig. 5E) and mineralized nodule formation (Fig. 5G).

PHF20 increases the enrichment of H3K4 methylation in association with methyltransferase such as MLL1^16^. To further confirm the involvement of H3K4 in PHF20-induced osteogenesis, we also observed the effects of si-MLL1 treatment on ALP enzyme activity. As shown in Fig. 5H, treatment of si-MLL1 inhibited ALP activity as si-PHF20 did. These results indicate that PHF20 protein can regulate Runx2 expression via increasing methylation enrichment of specific histone residues at P1 promoter of Runx2 to stimulate osteoblast differentiation.

## Discussion

To the best of our knowledge, this is the first report that elucidates the functional role of PHF20 in regulating the activation of Runx2 and osteoblast differentiation. In this study, we found that the methyl lysine effector protein PHF20 could stimulate osteoblast differentiation via direct interaction with Runx2 to increase Runx2 activation and promoting methylation of lysine residues of histones at the promoter of Runx2 to increase its expression.

Previously, conventional PHF20 knock-out mice have shown perinatal lethality with loss of lumbar vertebra and developmental anomaly^15^. However, that study did not mention the underlying mechanism involved in skeletal anomaly induced by PHF20 KO. In the present study, we performed gain- or loss- of-function studies in osteoblast lineage MC3T3-E1 cells to elucidate the role of PHF20 in osteoblast differentiation. We found that PHF20 expression increased during osteoblast differentiation. Overexpression of PHF20 increased osteoblast specific marker gene expression, ALP activity, mineralization, and Runx2 expression. On the contrary, inhibition of PHF20 expression by si-RNA transfection technology produced the opposite effects. These findings consistently suggest that PHF20 might have a stimulatory role in osteoblast differentiation.

Runx2 is a critical transcription factor that regulates osteoblast-specific gene expression such as osteocalcin gene. The activity of Runx2 is precisely regulated by other interacting proteins^25^. PHF20 can also interact with other proteins to control cellular activity along with methylation and acetylation processes of proteins^19, 26^. For example, PHF20 can function as an effector of p53 methylation to stabilize and activate the protein^19^. Our results also showed that PHF20 could physically interact with Runx2 and enhance Runx2’s transcriptional activity for Og2, Alp, and Bsp genes. After treatment with BMP2 to increase Runx2 protein level, overexpression of PHF20 also enhanced Og2 promoter activity. These findings suggest that PHF20 can act as a functional regulator of Runx2 via protein-protein interactions. During osteoblastic differentiation from mesenchymal stem cells, epigenetic modification precedes elevated transcriptional activity of Runx2 P1 promoter. It has been reported that H3K4me3 is functionally coupled to Runx2 gene up-regulation^24^. PHF20 can lead to methylation of histone proteins such as H3K4, thus controlling the expression of several genes^16, 19^. Our gain- or loss-of-function studies showed that PHF20 could affect Runx2 expression (Figs 2A,B and 3A,B). Moreover, overexpression of PHF20 increased cellular level of H3K4me3 and H3K4me3 enrichment at the P1 promoter region of Runx2. These results support that histone methylation at the P1 promoter region of Runx2 plays a major role in the regulation of Runx2 expression.

Bix-01294 is an inhibitor of histone methyltransferases. It can inhibit the methylation of several proteins in mammal cells and modulate epigenetic regulation of gene expression^20, 27^. In this study, Bix-01294 pre-treatment decreased the expression levels of H3K4me3 and Runx2, ALP activity, and mineralized nodule formation induced by PHF20. These results appear to come from the broad action of Bix-01294 because it can inhibit methylation events at a lot of protein sites^20, 27^. Reversely, addition of Ad-PHF20 following Bix-01294 increased the formation of mineralized nodules. This phenomenon supports the notion that PHF20-induced osteoblast differentiation may be related to methylation of histone protein. Overall, our findings indicate that PHF20 can stimulate osteoblast differentiation to increase Runx2 expression via increasing epigenetic methylation of histone at the P1 promoter of Runx2.

Although we examined the effects of PHF20 on osteoblast differentiation throughout this study, we do not exactly understand how PHF20 increased the level of H3K4me3 and Runx2 mRNA expression at a molecular level. PHF20 is a component of the MOF complex, which has acetyltransferase enzyme activity. We still need to further study on effects of PHF20 on acetylation of Runx2 or histone proteins. In addition, PHF20 can affect the action of MLL-KMT complex^16^. Involvement of MLL-KMT complex also has to be further examined in PHF20-induced Runx2 expression and osteogenesis.

In the present study, during the differentiation of MC3T3-E1 cells, PHF20 mRNA expression gradually increased up to 8 days after induction. Runx2 mRNA expression was peaked at 4 days without more increasing at 8 days (Fig. 1B). However, PHF20 overexpression increased levels of Runx2 and H3K4me3 proteins (Figs 2 and 5). Inferring from these findings, the increase in PHF20 expression at 8 days should have led to an increase in Runx2 expression, but Runx2 mRNA level was not increased. This phenomenon does not seem to be easily explained, but it might be possible in that PHF20 can complex with histone methyltransferase (MLL1) to regulate the transcription of genes^16, 28^. Even if the expression level of PHF20 is high, lack of its partners such as MLL1 may not permit PHF20 to increase the methylation of histone protein and subsequent gene expression. Additionally, we observed in the study that treatment of si-MLL1 inhibited ALP activity, as si-PHF20 did (Fig. 5H). These results suggest that PHF20 complicatedly might regulate Runx2 expression and osteogenesis through H3K4 methylation (Fig. 5I). Extensive and precise studies on the relationship between H3K4 methylated/acetylated osteogenesis and PHF20/MLL1/MOF complex are still needed.

Recently, epigenetic regulators of gene including histone methyltransferase and histone deacetylase have been considered as targets for the drug development^29^. The HDAC inhibitor Panobinostat has been approved by the FDA for the treatment of multiple myeloma, and the DNA methyltransferase inhibitor Decitabine for the treatment of myelodysplastic syndrome^30, 31^. Other HDAC inhibitors including MS-275 are being investigated for use in the treatment of bone disease^32^. In this study, we demonstrated that PHF20 epigenetically regulates Runx2 expression through H3K4 methylation and subsequently stimulates osteoblast differentiation. These findings provide evidence that PHF20 could be a good molecular target for controlling bone diseases.

## Materials and Methods

### Reagent and antibodies

Ascorbic acid-2-phosphate (AA) and β-glycerophosphate (β-GP) were purchased from Sigma-Aldrich Co. (St. Louis, MO, USA). Bone morphogenetic protein-2 (BMP2) was purchased from CowellMedi Corp (Seoul, Korea). PHF20 short interfering RNA (PHF20 si-RNA) was purchased from Santa Cruz Biotechnology (Dallas, TX, USA). Commercial antibodies against PHF20, Runx2 (Cell Signaling Technology, Beverly, MA, USA), Histone H3 (H3), Histone H3 Tri methyl K4 ChIP grade (H3K4me3), Sp7 (Osx) (Abcam, Cambridge, UK), β–actin (Santa Cruz Biotechnology, Dallas, TX, USA), and Myc (Thermo Fisher Scientific, Waltham, MA, USA) were used.

### Plasmids and adenoviruses

Reporter constructs containing mouse Osteocalcin gene 2 promoters-Luciferase (Og2-Luc) construct was kindly provided by Dr. Renny T Franceschi (University of Michigan School of Dentistry, Ann Arbor, MI, USA). Full length PHF20 (Flag-PHF20) and adenovirus PHF20 (Ad-PHF20) were previously described^26^.

### Cell culture, transient transfection, and viral infection

Murine pre-osteoblastic MC3T3-E1 cells and pluripotent mesenchymal lineage C3H10T1/2 cells were seeded at a mean density of 20,000 cells/cm^2^. Cells were cultured in α-minimal essential medium (Invitrogen) supplemented with 10% fetal bovine serum (FBS; Invitrogen) and antibiotics in a humidified atmosphere containing 5% CO_2_ at 37 °C. Osteoblast differentiation was induced by adding osteogenic medium containing 10% FBS, 50 μg/mL ascorbic acid, 5 mM β-glycopyrophosphate, and 100 μg/mL BMP2. Culture medium was replaced every 3 days. Transient transfections and viral infection were performed as described previously^23, 33^.

### Silencing of PHF20

For si-RNA transfection experiments, MC3T3-E1 cells were seeded into 6-well cell culture plates and transfected with PHF20 si-RNA (sc-152213, Santa Cruz Biotechnology) using Lipofectamine RNA iMAX reagent (Invitrogen) 24 hours later according to manufacturer’s instructions. Transfected cells were incubated for the indicated time followed by various analyses.

### Luciferase assay

MC3T3-E1 cells were transfected with indicated plasmids, including Og2, Alp, or Bsp gene promoters-luciferase reporters, Myc-tagged mouse Runx2, pcDNA3.1 (as an Empty Vector), and cytomegalovirus (CMV) β-galactosidase plasmid as an internal control using Lipofectamine2000 Reagent (Invitrogen) according to the manufacturer’s instructions. To measure promoter activity, cells were harvested at 48 hours post transfection. Luciferase activity was measured with a multi-plate reader (Bio-Tek Instruments) using luciferase reporter assay system (Promega). Luciferase activity was normalized to β-galactosidase activity.

### RT-PCR and quantitative real-time PCR analyses

Total RNA was extracted with TRIzol (Invitrogen) according to the manufacturer’s protocol. cDNA was synthesized from equal amounts of total RNA (2 μg) using random primers (Promega, Fitchburg, WI, USA) and Moloney Murine Leukemia Virus reverse transcriptase (MMLV-RT) (Promega). RT-PCR was performed at 37 °C for one hour followed by incubation at 72 °C for 15 min. PCR was performed with the following parameters: initial denaturation at 95 °C for 5 min followed by three-step cycling (25~30 cycles) of denaturation at 95 °C for 30 sec, annealing at 55 °C for 30 sec, and extension at 72 °C for 30 sec. PCR reactions underwent a final extension at 72 °C for 5 min. qRT-PCR was performed with ABI Step One Plus (Applied Biosystems, Foster City, CA, USA) using Quanti Mix SYBR Kit (Qiagen, Valencia, CA, USA) according to the manufacturer’s protocol. Data are presented as relative mRNA level of the gene of interest normalized to the mRNA level of endogenous β-actin. Relative target gene expression was quantified using comparative CT method. Primer sequences are listed in Supplementary Table 1.

### Western blot analysis

Total cell extracts were harvested in cell lysis buffer (Cell Signaling Technology) and centrifuged at 12,000 × g for 15 min at 4 °C. Quantification of total protein was performed using DC Protein Assay kit (Bio-Rad Laboratories, Hercules, CA, USA). Equal amounts of proteins were resolved by 8~12% sodium dodecyl sulfate - polyacrylamide gel electrophoresis (SDS-PAGE) and transferred to polyvinylidene difluoride (PVDF) membrane. After blocking with Tris-buffered saline with 5% milk and 0.1% Tween-20, the membrane was incubated with antibodies specific for PHF20 (#3934, Cell Signaling Technology), Runx2 (#8486, Cell Signaling Technology), H3K4me3 (ab8580, Abcam), H3 (ab1791, Abcam), OSX (ab187158, Abcam), Myc (MA1-21316, Thermo Fisher Scientific), and β-actin (sc47778, Santa Cruz Biotechnology). Signals were visualized using an enhanced chemiluminescence (ECL) reagent (Santa Cruz Biotechnology) in a LAS-4000 Lumino image analyzer system (Fujifilm, Tokyo, Japan).

### Alkaline phosphatase activity and alizarin red staining

For mineralization analysis, MC3T3-E1 cells were cultured with AA (50 μg/ml), β-GP (5 mM), and BMP2 (100 ng/ml) for 3 days for ALP stain or 8 days for alizarin red staining. For alkaline phosphatase enzyme activity, cultured cells were fixed with 4% formaldehyde (Sigma-Aldrich), rinsed three times with deionized water, and treated with 5-bromo-4-chloro-3-indolyl phosphate (BCIP^®^)/nitro blue tetrazolium (NBT) Liquid Substrate solution (Sigma-Aldrich) for 15 min in a dark room. Stained culture plates were scanned with a HP Officejet Pro L7580 scanner (HP Korea, Seoul, Korea). To evaluate mineralization, alizarin red staining was performed. Cells were fixed with ice-cold 70% ethanol for one hour, washed with cold deionized water, and then treated with 40 mM of alizarin red solution (pH 4.2) for 15 min at room temperature. After washing with PBS, stained cultures were then photographed. For quantitative analysis, ALP stained cells were quantified using ImageJ software (National Institutes of Health). Alizarin red stained cells were extracted with 10% cetylpyridinium chloride in 10 mM sodium phosphate for 15 min. Staining was quantified by measuring the absorbance at wavelength of 562 nm using a multi-plate reader/spectrophotometer (Multiscan Go, Thermo Fisher Scientific).

### Immunoprecipitation and chromatin immunoprecipitation assays

HEK-293T cells were transfected with indicated constructs for 48 hours. MC3T3-E1 cells were treated with BMP2 for 72 hours. Cells were harvested with cell lysis buffer (Cell Signaling Technology) containing protease inhibitors (Roche, Basel, Switzerland). Samples were centrifuged at 12,000 × g for 15 min at 4 °C and supernatants were pre-cleared with protein G–agarose beads (Invitrogen) prior to overnight incubation with anti-PHF20 (Cell Signaling Technology) and anti-Myc antibodies (Thermo Fisher Scientific). Protein G–agarose beads were added to cell lysates, incubated for 4 hours, washed 5 times with lysis buffer, and re-suspended in SDS sample buffer. After samples were resolved by 8% SDS-PAGE, Western blot analyses were performed using designated antibodies. ChIP assay was performed as described previously^24^. MC3T3-E1 cells cultured in growth medium or osteogenic medium were transfected with indicated constructs for 48 h and fixed with 1% formaldehyde for 10 min at room temperature. After washing with 10 ml of PBS three times, double cross-linking was performed using ethylene glycol-bis (succinic acid N-hydroxysuccinimide ester (EGS, Sigma) to have strong coupling with chromatin modifying enzyme^24, 34^. Cells were incubated with EGS for 1 hour at room temperature, washed three times with cold PBS, harvested, and sonicated in cell lysis buffer (Millipore Corporation, Billerica, MA, USA). Chromatin was sheared to obtain fragments of 500 bp or smaller. Soluble chromatin was subjected to immunoprecipitation. DNA fragments were then recovered by phenol/chloroform extraction and ethanol precipitation. Quantitative PCR analysis was then performed for Runx2 P1 promoter region with specific primer set; forward, 5′-GTG GTA GGC AGT CCC ACT TT-3′; reverse, 5′-TGT TTG TGA GGC GAA TGA AG-3′)^24^.

### Statistical analysis

All experiments were repeated at least three times. All values were expressed as means ± SEM of 3-independent samples. Data sets that passed the normality test with Shapiro-Wilk test were further analyzed with one-way ANOVA with Dunnett’s post-hoc test for multiple comparisons. Differences between groups were considered significant at p < 0.05. All analyses were performed using Prism Software (GraphPad Software Inc., San Diego, CA, USA).

## Electronic supplementary material

Supplementary Information
