# Supplementary material for: PHF20 positively regulates osteoblast differentiation via increasing the expression and activation of Runx2 with enrichment of H3K4me3
Source: Sci Rep. 2017 Aug 14;7:8060. doi: 10.1038/s41598-017-08868-0 (PMC5556080; doi:10.1038/s41598-017-08868-0)
Supplement: Supplementary file 1 — Supplementary Information [file 41598_2017_8868_MOESM1_ESM.doc]

**PHF20 positively regulates osteoblast differentiation via increasing the expression and activation of Runx2 with enrichment of H3K4me3**

Jin-Woo Yang^1,2^, Byung-Chul Jeong^1,2^, Jongsun Park^3^, Jeong-Tae Koh^1,2^,*

^1^Department of Pharmacology and Dental Therapeutics, School of Dentistry, Chonnam National University, Gwangju, 61186, South Korea

^2^Research Center for Biomineralization Disorders, School of Dentistry, Chonnam National University, Gwangju, 61186, South Korea

^3^Department of Pharmacology and Medical Science, College of Medicine, Chungnam National University, Daejeon, 35015, South Korea

*Correspondence and requests for materials should be addressed to J.-T.K. (email: jtkoh@chonnam.ac.kr)

Supplementary information includes:

Supplementary Fig. 1

Supplementary Fig. 2

Supplementary Table. 1


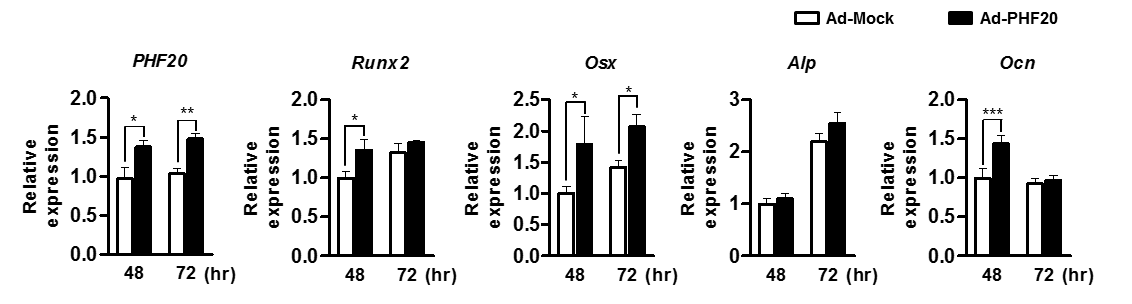


**Supplementary Figure 1. Long-term expression patterns of osteoblast-specific genes following overexpression of PHF20.** MC3T3-E1 cells were cultured with osteogenic medium, including Ad-PHF20, for 72 hours, and then qRT-PCR was performed. Results are expressed as fold activity relative to the control. Values are means ± SEM (n = 3). *p < 0.05, **p < 0.01 and ***p < 0.001 compared to the indicated group.


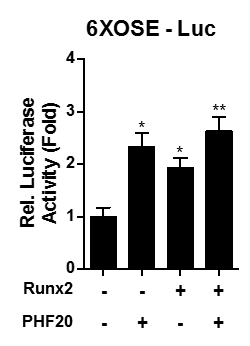


**Supplementary Fig. 2. Overexpression of PHF20 enhanced luciferase activity of 6xOSE promoter.** MC3T3-E1 cells were transiently transfected with 6x OSE-Luc (200 ng) plasmid with Runx2 (+, 100 ng) and/or PHF20 (+, 200 ng) construct. Forty eight hours after transfection, luciferase assay was performed as in “Materials & Methods”. Results are expressed as fold activity relative to the control. Values are means ± SEM (n = 3). *p < 0.05, **p < 0.01 compared to the indicated group.

**Supplementary Table 1. List of primers** used for PCR and qRT-PCR

| **PCR** |  | **Primer sequence** |
| --- | --- | --- |
| *PHF20* | forward | 5′-ACCAAGCACCCACCTAACAG-3′ |
|  | reverse | 5′-AAAGGGCGCAGATAAGGACT-3′ |
| *Runx2* | forward | 5′-GAGGGCACAAGTTCTATCTG-3′ |
|  | reverse | 5′-CGCTCCGGCCCACAAATCTC-3′ |
| *Osx* | forward | 5′-TGAGGAAGAAGCCCATTCAC-3′ |
|  | reverse | 5′-ACTTCTTCTCCCGGGTGTG-3′ |
| *Alp* | forward | 5′-TGAGGAAGAAGCCCATTCAC-3′ |
|  | reverse | 5′-ACTTCTTCTCCCGGGTGTG-3′ |
| *Alp (For qRT-PCR)* | forward | 5′-TTTCCCGTTCACCGTCCAC-3′ |
|  | reverse | 5′-ATCTTTGGTCTGGCTCCCATG-3′ |
| *Ocn* | forward | 5′-CTCCTGAGAGTCTGACAAAGCCTT-3′ |
|  | reverse | 5′-GCTGTGACATCCATTACTTGC-3′ |
| *Ocn (For qRT-PCR)* | forward | 5′-GCAATAAGGTAGTGAACAGACTCC-3′ |
|  | reverse | 5′-GTTTGTAGGCGGTCTTCAAGC-3′ |
| *β-actin* | forward | 5′-TTCTTTGCAGCTCCTTCGTTGCCG-3′ |
|  | reverse | 5′-TGGATGGCTACGTACATGGCTGGG-3′ |
| *β-actin (For qRT-PCR)* | forward | 5′-ACCCACACTGTGCCCATCTAC-3′ |
|  | reverse | 5′-GCCATCTCCTGCTCGAAGTC-3′ |
